# Bioprinting in Microgravity

**DOI:** 10.1021/acsbiomaterials.3c00195

**Published:** 2023-05-08

**Authors:** Misagh Rezapour Sarabi, Ali K. Yetisen, Savas Tasoglu

**Affiliations:** † Mechanical Engineering Department, School of Engineering, Koç University, Istanbul, Turkey 34450; ‡ Physical Intelligence Department, Max Planck Institute for Intelligent Systems, Stuttgart, Germany 70569; § Department of Chemical Engineering, Imperial College London, London SW7 2AZ, U.K.; ∥ Koç University Translational Medicine Research Center (KUTTAM), Koç University, Istanbul, Turkey 34450; ⊥ Koç University Arçelik Research Center for Creative Industries (KUAR), Koç University, Istanbul, Turkey 34450; # Boğaziçi Institute of Biomedical Engineering, Boğaziçi University, Istanbul, Turkey 34684

**Keywords:** 3D bioprinting, microgravity, space exploration, tissue engineering, regenerative medicine

## Abstract

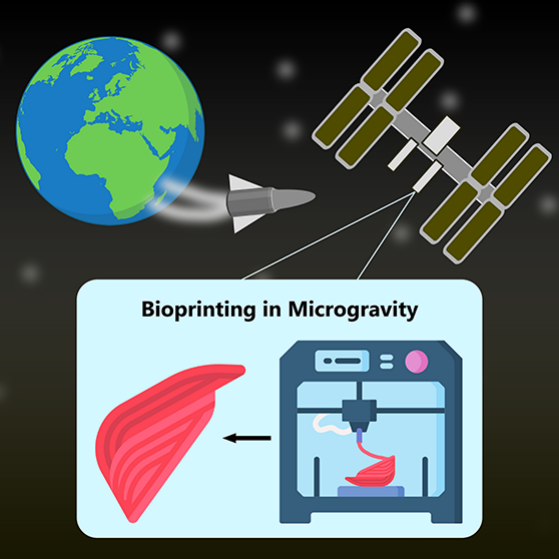

Bioprinting as an extension of 3D printing offers capabilities for printing tissues and organs for application in biomedical engineering. Conducting bioprinting in space, where the gravity is zero, can enable new frontiers in tissue engineering. Fabrication of soft tissues, which usually collapse under their own weight, can be accelerated in microgravity conditions as the external forces are eliminated. Furthermore, human colonization in space can be supported by providing critical needs of life and ecosystems by 3D bioprinting without relying on cargos from Earth, e.g., by development and long-term employment of living engineered filters (such as sea sponges–known as critical for initiating and maintaining an ecosystem). This review covers bioprinting methods in microgravity along with providing an analysis on the process of shipping bioprinters to space and presenting a perspective on the prospects of zero-gravity bioprinting.

## 1 Introduction

Bioprinting is an extension of traditional 3D printing, functioning on the same core additive manufacturing processes, in which material is appended to the print in cumulative layers to shape 3D products.^1−3^ Rather than printing with resin or thermoplastics, bioprinters are designed for compatibility with cell-laden bioinks.^4,5^ Bioprinters utilize various bioinks, including extrusion-based printing using filaments, laser-assisted bioprinting, and inkjet printing of liquid droplets.^6^ Through the advances in materials especially polymers,^7−13^ 3D printing has been established in biomedical applications.^9,14−18^ Bioprinting has also many tissue engineering and regenerative medicine applications, including, but not limited to, organ-on-a-chip devices for medical and pharmaceutical research^19−22^ and *in vitro* models of disease tissues such as tumors for cancer research,^23^ human tissue regeneration such as bone, skin, blood vessels, cartilage, and even internal organs to replace those which are damaged or diseased,^4,24−30^ stem-cell research,^31−33^ and organoid creation.^34^ 3D-bioprinting can enable producing complex 3D structures to mimic the *in vivo* microenvironment.^35−38^ With a growing request for scaled-up readily available biomimetic organs and tissues, advances in bioprinting technologies are increasingly imminent and necessary to provide high-throughput, precise construction of cell-laden structures.^39−42^

**Figure 1:**
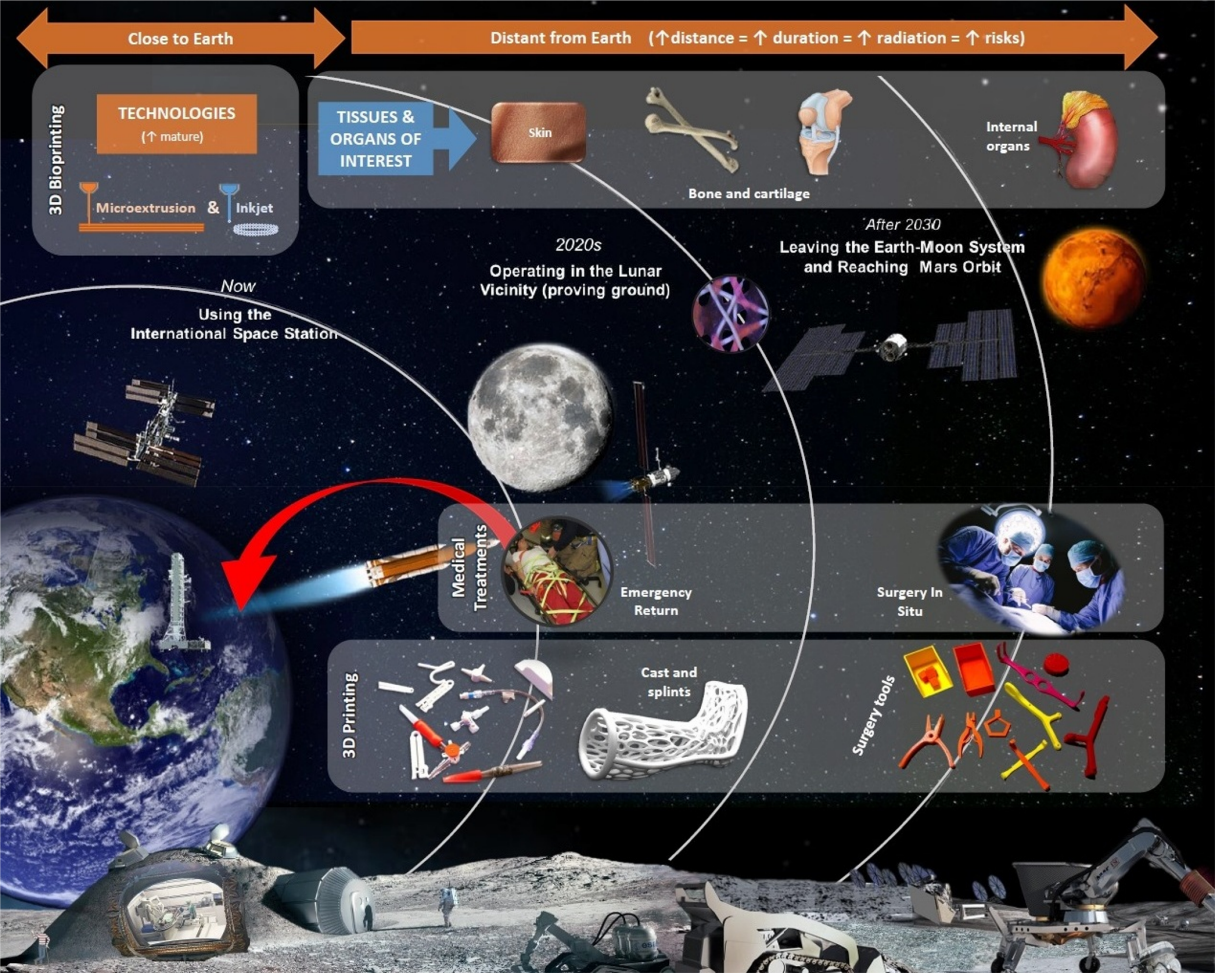
Bioprinting in microgravity: Fabricating using bioprinting technology by shipping bioprinters into space where the gravity is zero can be beneficial in several ways: First, fabrication of soft tissues which usually collapse under their own weight can be accelerated in the microgravity conditions as the external forces are eliminated, resulting in advancing the tissue engineering applications. Second, human colonization in space can be supported by providing critical needs of life and ecosystems without relying on cargos from Earth, which is the potential to be the next step to start life on other planets. Adapted with permission, copyright 2018 OHB SE. The composition of the figure was done by OHB (ohb.de) with images from the European Space Agency (ESA) and the National Aeronautics and Space Administration Agency (NASA).

Bioprinting is a high-impact, transformative biomedical technology that is consistent with the goals of National Aeronautics and Space Administration agency (NASA), Center for the Advancement of Science in Space (CASIS) initiative,^43^ and other space agencies. For example, CASIS offers access to the ISS along with experimental variables of microgravity settings for stem cell research.^44^ Bioprinting enables construction of models for normal and pathological living systems for the development and testing of medical interventions. It also enables the design and fabrication of systems that include both living and nonliving components for medical use. Bioprinting will have a long-term impact in tissue engineering and regenerative medicine.^45^ Printing soft and easily flowing biomaterials allows for mimicking the natural conditions of the human body;^46−48^ however, these prints usually collapse even under their own weight.^49−53^ One way to retain shapes in such situations is printing in very small gravity. Hence, the ability to conduct bioprinting research on the International Space Station (ISS) will provide greater knowledge and capabilities of the biomanufacturing of 3D tissues and organs. For instance, the microgravity environment of the ISS can be leveraged to explore scaffold-free 3D bioprinting. Although scaffold-free tissue engineering has been demonstrated in ground-based experiments, it has been limited in its scope and applicability to different cell types.^54^ Additionally, there is usually a need for keeping the printed tissue in the microgravity conditions for a couple of weeks until it strengthens by self-culturing. Scaffold-free bioprinting in microgravity would enable the exploration of how printed cells interact with each other in the absence of excess material. The advantages of microgravity could also enable the 3D bioprinting of complex structures and tissues that remain more difficult to fabricate in ground-based experiments due to limitations of the need for structural support. Furthermore, bioprinting can also benefit space exploration and research with ways to engineer artificial integrants to support human life and to enable the colonization of space. For instance, long-term development and employment of living engineered filters (such as sea sponges) can be a key development in resource-limited settings due to limited carbon (organic matter), nutrients, and minerals.^55^ This strategy would allow for the collection of resources critical for life supporting ecosystems, instead of heavily relying on cargo from the Earth. Living filters are a must to start and/or support an ecosystem—one that is natively an essential desert—for the exploration and eventual colonization in space.^55,56^ Importantly, these provide Earth-independent, life-sustaining filtering capabilities. The most promising tool to create useful objects such as living filters in resource-limited environments (i.e., space) is 3D printing. 3D printing offers the ability to customize objects and produce them in a limited space with limited materials. Herein, bioprinting studies carried out in microgravity simulated conditions are reviewed along with feasibility analysis for shipping bioprinters to space to provide a perspective on the missions of advancement in soft tissue science and starting ecosystems on other planets to pioneer this interdisciplinary area and shift the paradigms with advances in the multidisciplinary sciences (Figure 1).

## 2 Main Considerations for Feasibility

Since gravity in other planets of the solar system is always present, the most extreme case is the no-gravity condition in space beyond the planets. Additionally, studying printing in microgravity has importance in terms of research on fabrication of tissue/organ models. Fabrication of soft tissues, which usually collapse under their own weight during the printing process, can be considered as an accelerating factor (as the first intuition) in microgravity settings. In the meantime, the bioprinting process is a complex phenomenon encompassing dynamics interactions between deposited layers and upcoming layers. The resulting shape of a deposited droplet or filament (depending on the printing mode) and its tunable material properties via cross-linking affect the boundary conditions of the oncoming layers’ deposition or droplets’ impact. Therefore, there is a growing need to resolve the complex fluid dynamics with regards to varying microgravity conditions in order to achieve overall shape fidelity of the final constructs to advance 3D bioprinting. For instance, cross-linking processes can be controlled and applied at different phases, such as during the cell-laden droplet flight from the nozzle or spreading of the cell-laden droplet during the impingement onto a receiving surface. The onset time of cross-linking affects and dynamically alters the material properties of the encapsulating liquid, including density, viscosity, and viscoelastic features, and eventually affects the complex fluid dynamics of the encapsulating droplet and shear rates experienced by the cell and the final shape of the deposited droplet. In this section, the main considerations for feasibility are presented.

### 2.1 Transportation of Bioprinters

The process of shipping bioprinters to space for tissue engineering research faces some important challenges. First, the expenses related to the whole process should be considered. Second, the functionality of the bioprinter device itself under microgravity conditions must be studied. In this section, these challenges are explained.

**Figure 2:**
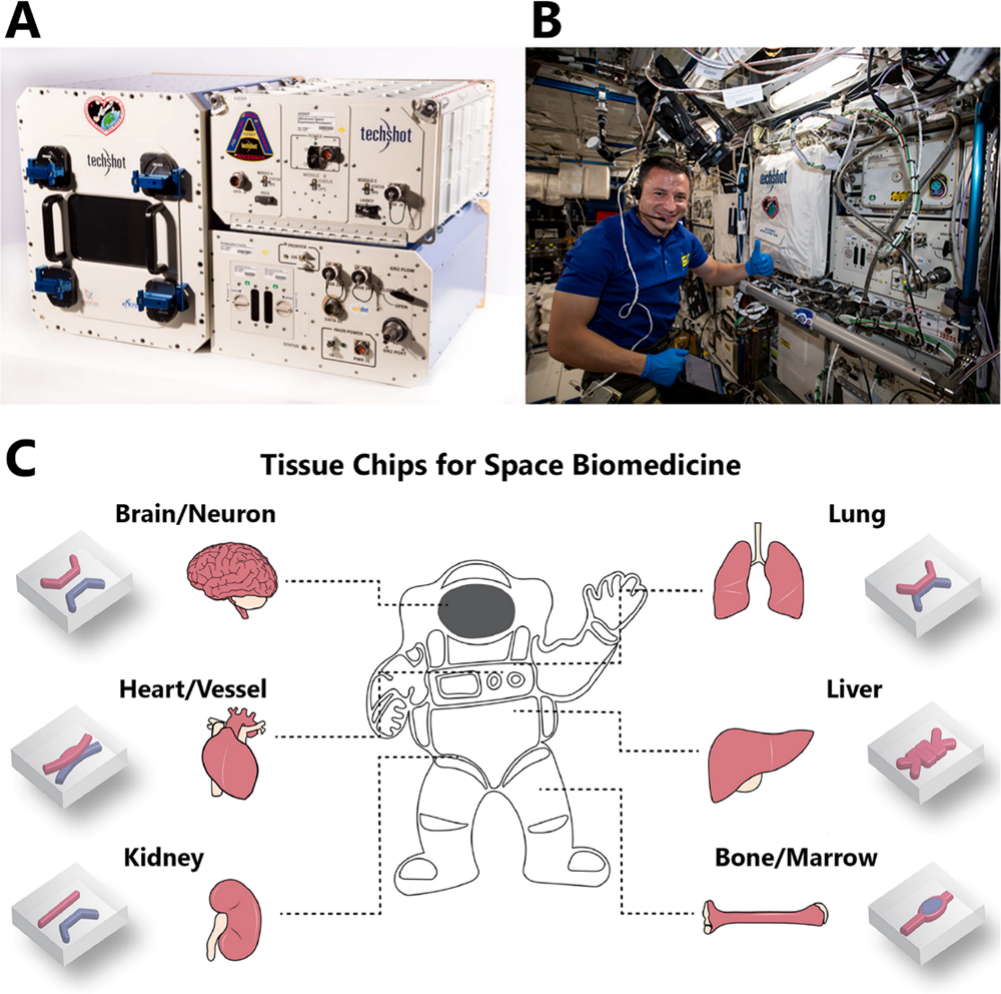
The 3D BioFabrication Facility (BFF) developed by Techshot. (A) BFF consists of the print volume on the left part and the power and data handling module in the lower right. The top right box is a separate device called the ADvanced Space Experiment Processor (ADSEP), where the cells printed with BFF are conditioned into tissue. Adapted from the Web site of Techshot (techshot.com) with permission, copyright 2019 Techshot. (B) BFF has already been shipped to the International Space Station (ISS) for studying bioprinting in microgravity. Adapted from the Web site of the ISS National Lab (issnationallab.org) with permission, copyright 2019 ISS National Laboratory. (C) Schematics view of tissue chips for recapitulating several tissue-level physiological functions, aiming for space medicine applications. Adapted from ref (81), copyright 2022 Elsevier, in accordance with Creative Commons CC-BY-NC-ND license.

NASA sent the first 3D printer to the ISS in 2014.^57^ Developed by “Made in Space” company, the 3D printer runs based on the technology of the fused filament fabrication (FFF) process.^57^ In addition, NASA has sent another 3D printer to the ISS, as part of The Redwire Regolith Print (RRP) mission.^58^ The goal of the RRP mission is 3D printing in space using simulated lunar regolith as feedstock material, paving the way for the next mission of NASA: the Artemis program, with the goal of landing the first female astronaut and next male astronaut on the Moon.^59^ Furthermore, the 3D BioFabrication Facility (BFF) developed by Techshot has already been shipped to the ISS (Figure 2).^60^ To transport the bioprinter to the ISS, the cost of the payload to reach low Earth orbit (LEO) must be considered. While the cost of shipping to LEO is not directly available, estimates can be calculated using resources such as the U.S. Federal Aviation Administration reports on The Annual Compendium of Commercial Space Transportation.^61,62^Table 1 shows the mentioned costs in United States Dollars (USD) per kilogram for several spacecrafts. In addition, the expenses related to the scientist or operator that is going to work with that printer should be considered as well. Besides preparation costs, ticket prices for human spaceflight vary from $95k to $250k per seat.^61^

**Table 1:** Cost Estimation for the Payload of Several Spacecrafts to Reach Low Earth Orbit (LEO) from Ground in USD per Kilogram^b^

**Spacecraft name**	**Cost of shipping to LEO (USD/kg)**	**Ref**
SpaceX Falcon 9	∼4,100 to 4,600	(61, 62, 94)
CALT Long March 3B	∼6,400^a^	(95)
RSC Energia Soyuz	∼7,700^a^	(95)
Orbital ATK Antares	∼11,400 to 23,000	(61, 62)
ULA and LMCLS Atlas V	∼12,000 to 13,500	(61,62)
Arianespace SA Ariane 5	∼13,200^a^	(95)
ULA Delta IV	∼13,800 to 17,400	(62)
Orbital ATK Minotaur-C	∼30,000 to 34,300	(61, 62)
Orbital ATK Pegasus XL	∼30,000 to 89,000	(61, 62, 95)
Rocket Lab Electron	∼1,000,000 to 2,700,000	(61)

a The prices indicated are adjusted for inflation for an equivalent price in 2023 as ref (95) reports prices from 2002. The calculation is based on an average inflation rate of ∼2% and cumulative inflation of ∼45% between 2002 to 2023.

b Sorted by increasing price.

3D bioprinters can be customized to have lower weights. The respective weights of the components of a custom bioprinter setup (17 kg)^63^ are reported and itemized in Table 2. Another custom bioprinter was reported to be around 3 kg.^64^ In addition to custom ones, the weights of some of the available 3D bioprinters are listed in Table 3. To reduce the total weight of a bioprinter, while also improving the overall fabrication and reproducibility of the setup, components of the bioprinter setup may be 3D-printed. High-end 3D printers, such as the Carbon M2 Printer, can be utilized to fabricate custom parts and components using various lightweight and high-strength materials. The Carbon M2 implements digital light synthesis (DLS) that uses digital light projection, oxygen permeable optics, and programmable liquid resins to produce parts similar in material properties and finish to injection-molded parts. After the printer uses light excitation to print and form the part, the part is subsequently baked to cause a secondary chemical reaction resulting in added strength. Printable materials include silicone, rigid, flexible, and elastomeric polyurethane, cyanate ester, epoxy, and urethane methacrylate. Rigid materials such as rigid polyurethane, cyanate ester, and epoxy can be used to fabricate portions of the bioprinter frame, print bed, syringe holder, syringe caps and lure locks, and the dispensing head and curing light holder.

**Table 2:** Respective Weights of the Components of a Custom Bioprinter Setup^63^

**Name**	**Weight (g)**
CNC frame kit with stepper motors	7652
MKS Base v1.5 controller	159
3.2′′ color touch screen	142
3.5 mm stereo connector	10
Illuminated buttons	70
4-channel MOSFET board	32
Metal enclosure	1317
Solid-state relay	18
DC power jack	45
Emergency stop button	10
6-pin connectors	204
Power socket	23
Power supply	953
UV led	715
Dispensing head	100
Coaxial needle (18G-22G)	400
Micro limit switches	20
Solenoid valve	23
Air manifold	100
Lead screw	113
Bearing bracket	77
Aluminum profiles	680
Flex coupling	23
Shaft	280
Linear bearing	136
T-slot nuts	207
Stepper motor	58
Unmeasured 3D printed parts	-
Total weight: 13,567 g	
**Total weight + 25% Error: ∼ 17 kg**	

**Table 3:** Weight of Several Commercial 3D Bioprinters

**Name of the 3D printer**	**Weight (kg)**	**Ref**
Techshot 3D BioFabrication Facility (BFF) along with ADvanced Space Experiment Processor (ADSEP)	172 + 23 = 195	Information from company
Carbon M2	317	Information from company
Carbon L1	800	Information from company
EnvisionTEC 3D Bioplotter	90 to 130 (depending on the series)	(96)
RegenHU R-GEN 100	160	(97)
CELLINK Bio X	18	(98)
CELLINK INKREDIBLE+	15	(99)

**Figure 3:**
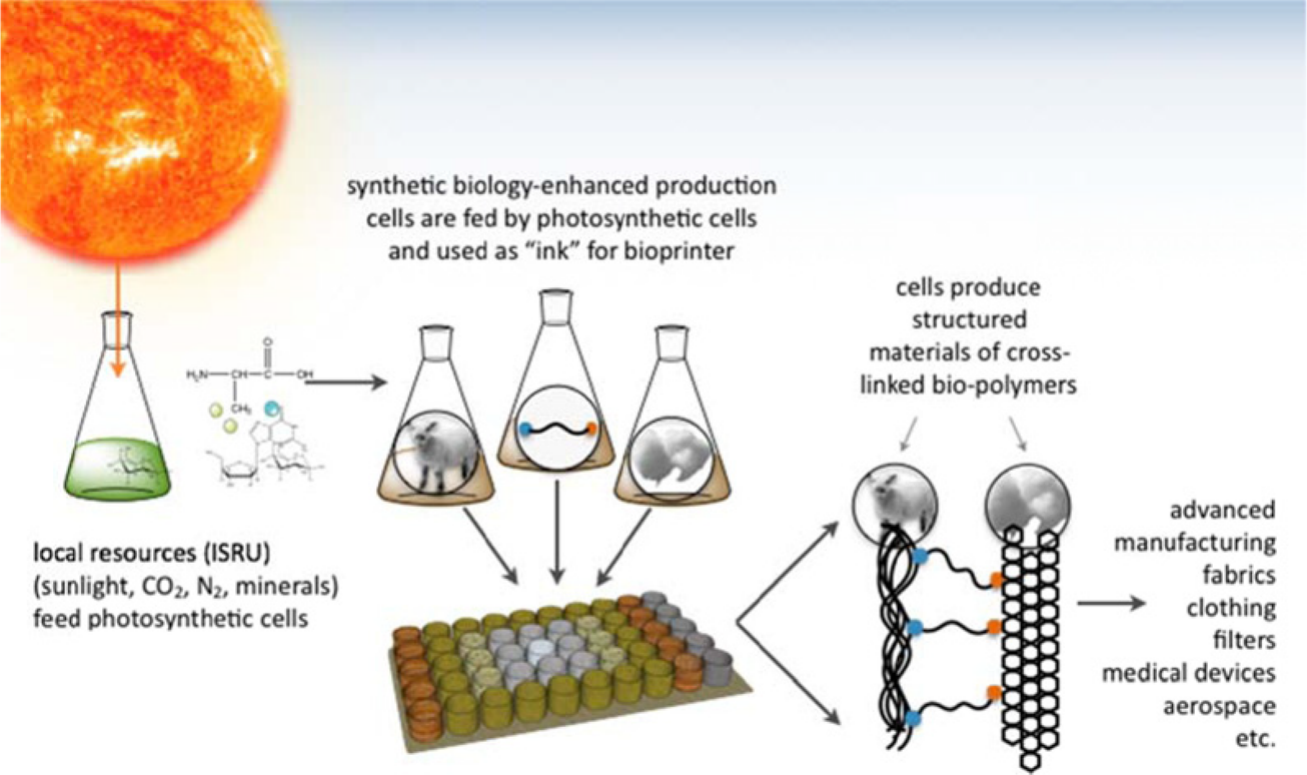
The launched material into the destination planet must be light, stable, and reliable. Synthetic biology science can play an important role in this regard by enhancing production cells which are utilized to be fed into photosynthetic cells from local resources and be used as the required bioink for the bioprinting process. This process provides a vision for a biology-based Mars colony. Adapted from ref (65), copyright 2016 Portland Press, in accordance with the Creative Commons Attribution (CC BY) license.

Sending materials into space should undergo several considerations to maximize the efficacy. The launched material must be light (to increase the cost efficiency) and stable (to be reliable at the landing place).^65^ An example of the importance of this point can be seen in the robots that are sacrificed, because of not only protection from severe radiations but also for saving costs. Synthetic biology science can enable materials that solve these challenges (Figure 3). In this regard, synthetic biology-enhanced production cells are utilized to be fed into photosynthetic cells from local resources and be used as the required bioink for the bioprinting process.^65^

### 2.2 The Effects of Microgravity on the Performance of Bioprinters

3D bioprinting can be carried out mainly using inkjet-based, laser-assisted, and extrusion-based technologies.^30^ The effects of microgravity on the working mechanisms in each technology should be considered. In inkjet-based bioprinting, droplet impact physics can be affected due to less gravity. Nozzle clogging also can occur because of slower speed in cell-laden droplets falling onto the surface. Laser-assisted bioprinters would be least affected by microgravity as the laser beam is not affected negatively by microgravity. However, laser-assisted 3D bioprinters need post cleaning since the process has residue materials, while inkjet-based 3D bioprinters use all the formed droplets toward shaping the final construct. However, these two technologies are not the main players in bioprinting. Extrusion-based bioprinting has become a more popular technique in bioprinting because of being cost-efficient and offering precise control and resolution with high repeatability.^30,42,66,67^ In extrusion-based 3D bioprinters, the pressure by a piston or a pneumatic system extrudes the bioink through the nozzle of the printer to shape a continuous filament, which constructs the final structure. Due to this mechanism, these bioprinters can be used even in the upward direction against the gravity of the Earth. Two samples, alginate/methylcellulose hydrogel, one of the bioinks used in bioprinting, and calcium phosphate bone cement (CPC), one of the macroporous scaffolds used in bone tissue engineering, were reported to be printed in the upward direction,^68^ suggesting that these bioprinters can be used in zero-gravity conditions as well. In addition to zero-gravity conditions, temperature can also be another affecting parameter in this regard. However, the effect of temperature is usually more important for the product than the printer itself. While bioprinters provide the optimal temperature and cross-linking extent for the consumed ink in the procedure, the printed products that contain cells should be kept in temperatures with minimal risk to the encapsulated cells. Additionally, an experimental study reported the control of temperature of experimental containers that were launched to the ISS, delineating that the temperatures did not exceed 7.1 °C from preflight handover until storage after arrival at the ISS.^69^

## 3 Bottom-up Tissue Engineering in Engineered Microgravity Settings

Simulating effects of microgravity enables the opportunity to study the experiment or setup conditions for prognosticating their outcome and understanding the conceivable issues. Microgravity settings or weightlessness conditions can be achieved via different methods, including (i) clinostat, (ii) random positioning machine (RPM), (iii) rotating wall vessel (RWV), and (4) magnetic levitation.^70^

Clinostat setups rotate the study system perpendicular to the vector of gravity to make the gravitational acceleration ineffective. Random positioning machines (RPMs) rotate the study system around two rotational axes in a gimbal mount, with continuous random changes in the velocity and direction of rotation. Rotating wall vessels (RWVs) are completely filled with a fluid and work in a similar manner to clinostat setups by transferring rotational velocity onto the study system, making its components fall through circular pathways. Finally, magnetic levitation setups benefit from the magnetic field forces for enabling the levitation of the study system. This method is usually preferable for conducting microgravity research in comparison with the other techniques that employ mechanical rotation creating extra forces and stresses on the study sample.^70^

**Figure 4:**
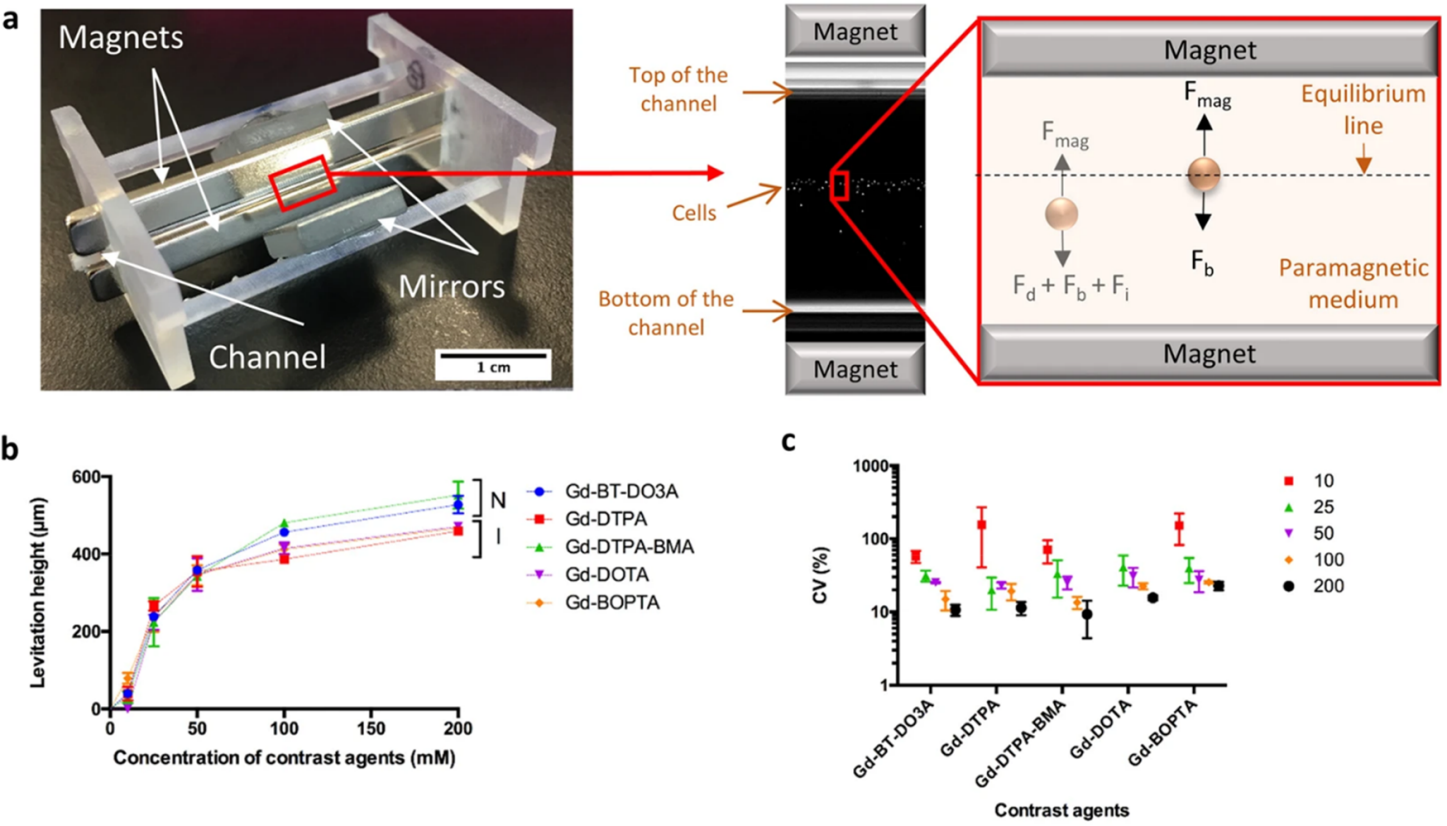
The magnetic levitation setup used for biofabrication of *in situ* self-assembled 3D cell cultures inside a microcapillary channel system. (a) Mirrors are positioned at 45° on two sides to help visualize the cells with microscopes. Neodymium magnets are positioned on the sides with negative poles facing the inner microcapillary levitation and culturing channels. (b) Plot of cells’ levitation heights for different Gd concentrations. (c) Coefficient of variation (CV%) of levitation heights for different solutions based on Gd (Gd-BT-DO3A, Gd-DTPA, Gd-DTPA-BMA, Gd-DOTA, and Gd-BOPTA). Abbreviations: N: nonionic agents, I: ionic agents, F_d_: fluidic drag force, F_i_: inertial force, F_b_: buoyancy force, and F_mag_: magnetic force. Scale bar: 200 μm. Adapted from ref (73), copyright 2018 Springer Nature, in accordance with the Creative Commons Attribution (CC BY) license.

Magnetic levitation utilizing negative magnetophoresis, also named diamagnetophoresis, can be used to set up the conditions of weightlessness to study its effects for several applications.^71^ Bioassembly of 3D constructs may additionally be promoted using magnetic levitation in microgravity studies for the fabricated 3D tissue structures. For example, a custom device was designed and developed in this regard for fabricating 3D cartilage tissue structures in the ISS.^72^ In other research, a 3D cell culture with the self-assembly ability *in situ* was biofabricated in microgravity mimicking the situation created with magnetic levitation using gadolinium-based solutions (Figure 4).^73^ Considering cell viability factors and levitation location in the system, the most appropriate chelate form and gadolinium concentration for levitation and cell culture were determined. Short-term and long-term levitating *in situ* arrangements were then tested with different numbers of cells. Finally, various forms of biphasic cells were made from cancer cells and stem cells in a levitation system. This 3D cell culture study in the magnetic levitation system with real-time imaging was used to assess the effects of microgravity at the cellular and molecular levels.

**Figure 5:**
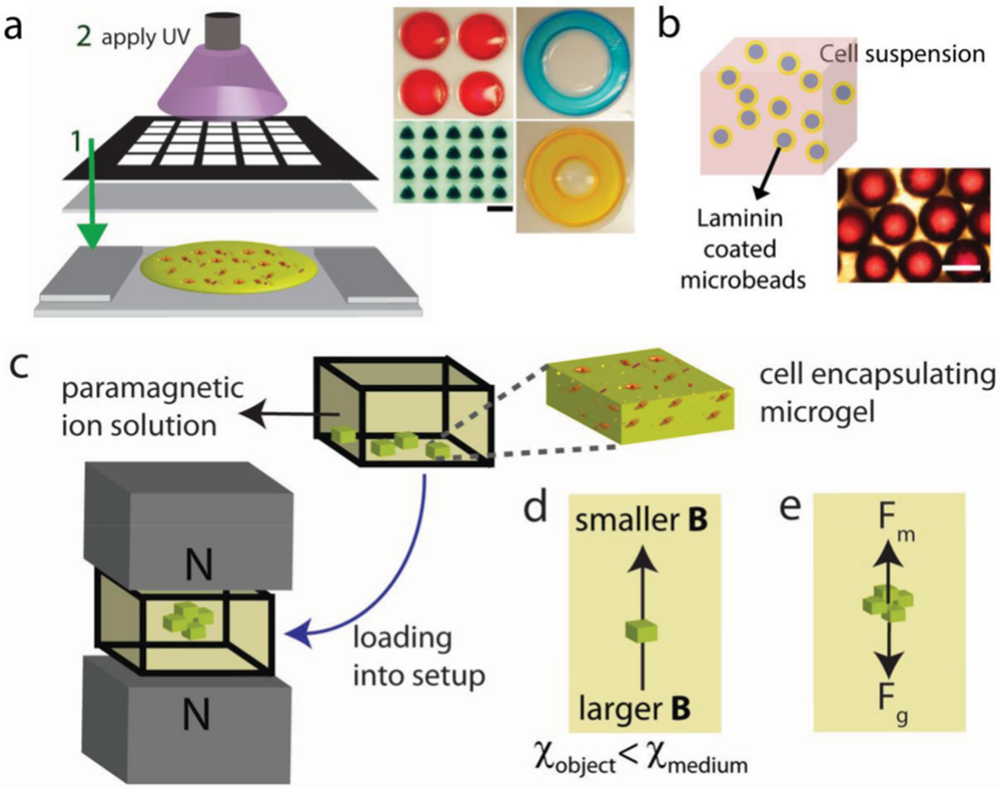
Magnetic levitational platform for soft living material fabrication. (a) Schematic view of fabricating units with photo-cross-linkable polymers using patterned mask photolithography. The glass sheets with a gel precursor solution were exposed to UV light (scale bar: 1 mm). (b) Fabrication of cell seeded microbead was performed by incubating laminin coated microbeads in cell suspension (scale bar: 500 μm). (c) Cell-encapsulating elements’ self-assembly in the magnetic levitational setup with two Neodymium magnets. (d) The particle moves from larger magnetic field strength (B) to lower magnetic field strength, if its magnetic susceptibility is lower than magnetic susceptibility of the suspending medium. (e) At equilibrium point, the two forces of magnetic (F_m_) and corrected gravitational (F_g_) (the difference between gravitational force and buoyancy force) act on the levitating particle. Adapted with permission from ref (74), copyright 2015 John Wiley & Sons.

The approach of magnetic levitation can also be used in fabricating living material.^74^ A solution containing paramagnetic characteristics was used for aligning different microstructures such as cell seeded microbeads or cell encapsulating hydrogels for 2D and/or 3D contact-free controlling of their assembly (Figure 5). Two Neodymium (NdFeB) magnets with the same poles facing each other were used to create the microgravity environment. Precise control and levitation of small particles were achieved by manipulating the extent between the magnets and diversifying the solution concentration used in the setup. With potential applications for bottom-up tissue engineering, this study presented a method for soft living material fabrication in weightlessness conditions.

Paramagnetic mediums, which are usually required for execution of magnetic levitation studies on the Earth, in the majority of the cases include gadolinium salts. Gadolinium is a highly toxic component,^75^ and this toxicity can be harmful to the nature of bioprinting setups, where bioinks (with laden cells) are used. A commentary about bioprinting in Russia has offered scrutinies of the related research, reviewing a magnetic levitational bioassembly setup, which was developed for fabrication of 3D tissue constructs in space under microgravity conditions from tissue spheroids made up of human chondrocytes, within paramagnetic solutions featuring low nontoxic concentrations.^76^ Bioassembled 3D tissue structures exhibited acceptable viability and advanced stages of the fusion process of tissue spheres. These results indicate that biofabrication using magnetic fields is a viable alternative to traditional methods based on scaffolds, labels, and nozzles. It opens a new perspective of research, promoting tissue engineering, space regenerative medicine, and the science of space life.

**Figure 6:**
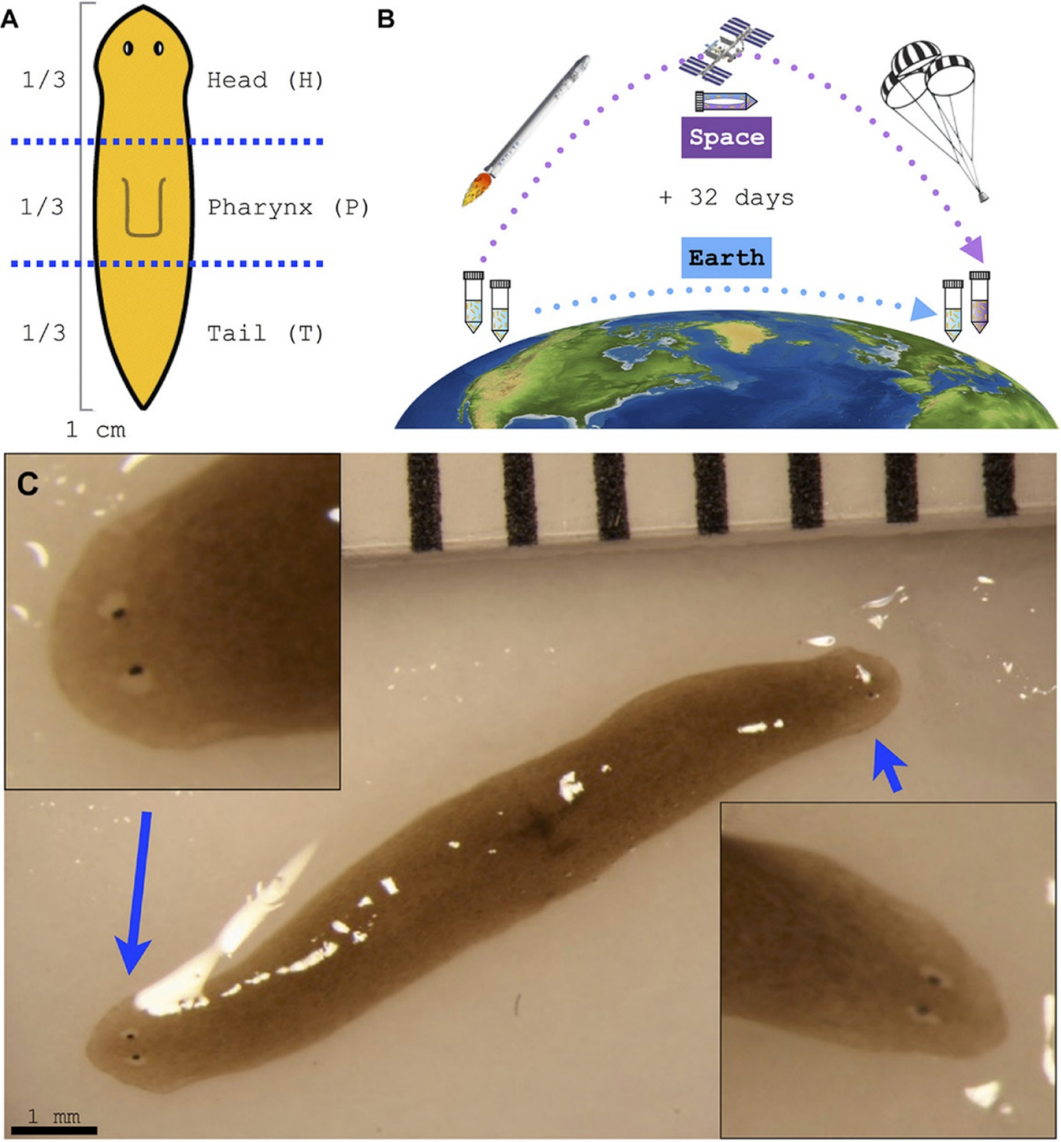
Planaria, well-known flatworms for regeneration studies, were sent to space to investigate their behavior under microgravity conditions. (A) Immediately upon separation of their heads and tails, (B) they were sent to the ISS, for a 32-day period, with an identical control group kept on Earth. After 32 days, the group in the ISS was sent back to the Earth. (C) In an exceptionally rare occurrence, one of the worms from space was regenerated into a phenotype with two heads. Adapted from ref (78), copyright 2017 John Wiley & Sons, in accordance with the Creative Commons Attribution (CC BY) license.

One of the well-known models for conducting tissue engineering and regeneration research is Planarian. Planaria are flatworms with the interesting ability to regrow all parts on their bodies, even if they are sliced into disparate head, pharynx, and tail fragments.^77^ To find answers for the question of how these worms behave in the absenteeism of gravity, they were sent to the ISS on 10 January 2015, for a 32-day period, immediately upon separation of their heads and tails (Figure 6).^78^ An identical group of Planaria worms was also kept under control on the Earth. Upon transportation of the space group back to the Earth, the two groups were analyzed. In an exceptionally rare occurrence, one of the worms from space was regenerated into a phenotype with two heads. In addition, this double-headed Planarian could regenerate into double-headed phenotypes again in plain water. Collectively, the discovered occurrence may be studied for triggering tissue regeneration.

Also other research in this regard reported 3D tissue culture based on magnetic cell levitation.^79^ The 3D culturing process was performed using a mixture containing a bioinorganic hydrogel, filamentous bacteriophage, nanoparticles of gold with the ability to self-assemble into the hydrogel, and magnetic iron oxide (MIO; Fe_3_O_4_, magnetite). Human glioblastoma cells were treated with this mixture and held at the interface of the air and medium using a magnet. The strategy for the cell levitation did not require scaffolds or matrices and included the following steps: (i) dispersion of hydrogel over the cells and incubation of the mixture, (ii) eliminating noninteracting sections of hydrogel, (iii) lifting the cells to the interface of air and medium using a magnet, and (iv) formation of characteristic multicellular structures after levitation for 12 h. Spatial control of the magnetic field enabled manipulation of the cell mass and the shape of the culture along with creation of multicellular clustering of various types of cells in the coculture. The observed protein expression for the levitated human glioblastoma cells was similar to the profiles for human tumor xenografts, indicating that the culture enabled by this levitation strategy intimately epitomizes the protein expression *in vivo* and has the potential to be utilized in biotechnology, drug discovery, stem cell research, or regenerative medicine.

A deeper understanding of the mechanism’s influencing wound healing capability in space is required since human skin is the tissue prone to damage during manned space missions. Long-term manned missions such as the exploration of Mars or other extraterrestrial planets and settlements will need the development of novel, efficient therapeutic solutions for serious skin injuries that are consistent with the constraints of the tools and supplies used on board spacecraft. 3D bioprinting is a promising technology to provide a solution to these demands. It permits the creation of multicellular, intricate, and three-dimensional tissue structures that may be used as models for fundamental research and as transplantable skin grafts.^80^ Utilizing bioprinting technologies for the fabrication of transplantable skin grafts provides several advantages over conventional approaches for tissue engineering. Bioprinting can be performed in an automated and semiautomated manner, which is efficient in reducing the operator’s need during space flight. Additionally, the fabrication process in bioprinting is time-efficient with less complexity compared to conventional methods, as cells and biomaterials are deposited simultaneously. Bioprinting provides a more precise control over the size and internal structure of the skin grafts, and optimal biomaterials can be selected for each cell type present in the procedure. Finally, 3D bioprinting allows for the fabrication of complex engineered tissues by integrating hair follicles, melanocytes, eccrine sweat and sebaceous glands, and cell types that support fast vascularization.

## 4 Future Perspective and Conclusion

Particularly on long-term missions such as Mars exploration, the environment of spaceflight, which includes microgravity and radiation exposure, can have a substantial impact on an astronaut’s health and performance. The numerous biological impacts of spaceflight have been studied using conventional experimental methods both on the Earth and in space. Microgravity and high-energy radiation, which can have extensive effects on biological systems and may result in pathological diseases, are the main reasons why the environment of spaceflight varies from that on the Earth. Microgravity can have an adverse effect on a variety of organs and tissues, including the immunological, musculoskeletal, renal, and cardiac systems. It can also cause physiological changes that might be harmful to an astronaut’s health and performance, such as loss of bone and muscle mass. This is because microgravity can affect blood flow, stem cell differentiation, and gene regulation in addition to being unable to supply the mechanical stimulation required for tissue homeostasis and regeneration. Challenges include the difficulties of simulating microgravity and space radiation on the Earth while researching the intricate biological impacts of spaceflight and creating space medicine. Tissue chips and organ-on-chip systems, bioengineered *in vitro* models, offer encouraging insights toward space medicine by recapitulating tissue-level physiology with a compact footprint and the potential for automation. Tissue chips are becoming increasingly important in space medicine, as seen by several tissue chips that have previously been flown into space.^81^ 3D bioprinting can be regarded as the next promising wave to engineer and improve human health, providing patient-specific print outputs for tissue engineering replacements using stem cells, with reduced risks of printed organs and tissues being rejected by the human body.^82,83^ Transplantation of organs from other humans is more subject to suffer from this issue; there may be ethical issues, but also it is hard to provide everyone with this opportunity as not every deceased person’s organs are usable.

Moreover, 3D bioprinting provides drug testing ability specific for each patient. All these benefits result in a more cost-efficient process in a long time since possible multiple transplantations, the antirejection drugs, and long conventional beforehand drug tests will no longer be needed. The challenges about expenses and functionality of bioprinters in microgravity should be further evaluated. In addition, effects of microgravity on the bones, muscles, tendons, ligaments, and soft tissues should be further analyzed to understand the possible limitations.^84^ The process of 3D bioprinting can be considered as an easily scalable method; however, it is not necessary for researchers to limit themselves only to the ISS. Designing microgravity laboratories that orbit the Earth can be an alternative path, similar to the thousands^85^ of orbiting satellites around the Earth. Additionally, applying procedures developed by Artificial Intelligence (AI) sciences such as deep learning and machine learning can also be beneficial in obliterating the need to send a human operator to space by delegating the associated responsibilities to the AI core, which can prepare and report faster analysis and forecast the expected results.^86−92^ Ultimately, a final challenge can potentially be that manufactured organs and tissues in space may require ethical approval and regulations.^93^
